# Preparation and Characterization of Salbutamol Sulphate Loaded Ethyl Cellulose Microspheres using Water-in-Oil-Oil Emulsion Technique

**Published:** 2010

**Authors:** Bipul Nath, Lila Kanta Nath, Bhaskar Mazumder, Pradeep Kumar, Niraj Sharma, Bhanu Pratap Sahu

**Affiliations:** a ***Department of Pharmaceutical Sciences, GIPS, Gauhati, Assam (N.E), India.***; b *** Department of Pharmaceutical Sciences, Dibrugarh University, 786004, Assam, India.***

**Keywords:** Salbutamol sulphate, Ethyl cellulose, Emulsion solvent evaporation method, Microspheres, Higuchi model

## Abstract

The aim of this study was to formulate and evaluate microencapsulated controlled release preparations of a highly water/soluble drug, salbutamol sulphate by (water in oil) in oil emulsion technique using ethyl cellulose as the retardant material. Various processing and formulation parameters such as drug/polymer ratio, stirring speed, volume of processing medium were optimized to maximize the entrapment. The release of salbutamol sulphate from ethyl cellulose microsphere was compared and possible release mechanism proposed. Microspheres were prepared by water in oil emulsion technique using acetonitrile/dichloromethane (1:1 ratio) solvent system. Span 80 was used as the dispersing agent and *n*-hexane was added to harden the microspheres. The prepared microspheres were characterized for their micromeritic properties and drug loading, as well as compatibility by infrared spectroscopy, differential scanning calorimetry (DSC), X-ray powder diffractometry and scanning electron microscopy (SEM). The *in-vitro *release studies were carried out in phosphate buffer at pH 7.4. The prepared microspheres were white, free flowing and spherical in shape. The drug-loaded microspheres showed 55.7 - 76.6 % of entrapment and release was extended up to 10 h. Various processing and formulation parameters such as drug/polymer ratio, stirring speed, volume of processing medium, etc. significantly affect the drug release from the microspheres. The best/fit release kinetics was achieved with Higuchi plot followed by zero order and first order. The release of salbutamol sulphate was influenced by altering the drug to polymer ratio and the drug release was found to be diffusion controlled.

## Introduction

Salbutamol sulfate is a short-acting beta-2 agonist (1) which is used to treat diseases such as asthma, emphysema and bronchitis. The plasma half-life of the drug has been estimated to range from 4 to 6 h, so the recommended dose in adults and children is usually given every 4 to 6 h (2). Because of its short biological half-life and low oral dose of 5 mg, salbutamol sulphate should be formulated in a sustained release dosage form to improve patient compliance (3, 4). Salbutamol sulphate is a highly water soluble drug, so microencapsulation of this drug provides the prolonged release of a single dose and thus minimizing frequent administration and reducing side effects.

There are several polymers reported for the microencapsulation of drugs using ethyl cellulose, cellulose acetate butyrate, cellulose acetate phthalate, polymethacrylates, polycaprolactone, etc. There is one literature found in which microspheres of salbutamol sulphate with poly lactic acid-co-glycolic acid (PLGA 85115) were prepared by the modified solvent evaporation method using a w/o/w double emulsion (5).

Ethyl cellulose is a water insoluble polymer and widely used in microencapsulation process even though it is non-biodegradable polymer due to its high safety, good stability, easy fabrication and cheapness. Several research workers have investigated the utilization of ethyl cellulose as a retardant polymer to encapsulate highly water soluble drug by emulsion solvent evaporation method (6, 7) and spherical crystallization technique (8).

The use of water in oil emulsion solvent evaporation technique is one method used to modify the drug release of highly water soluble drug. The use of w/o/w double emulsion method to microencapsulate salbutamol sulphate using poly (lactic acid-co-glycolic acid) as retardant polymer and PVA as emulsifying agent was reported (9). However, there was no such literature reported for the encapsulation of salbutamol sulphate by w/o/o method using ethyl cellulose as retardant polymer. 

Based on these considerations, the present work investigates the means for the efficient encapsulation of salbutamol sulphate into ethyl cellulose microspheres using w/o/o emulsion solvent evaporation method. Various process and formulation parameters such as drug/polymer ratio, stirring speed, volume of processing medium were optimized to maximize the entrapment. The release of salbutamol sulphate from ethyl cellulose microsphere was compared, and possible release mechanism proposed.

## Experimental

**Materials**

Salbutamol sulphate was obtained as a gift from Ducbill drugs, Kolkata, India. Ethyl cellulose (14 cps viscosity grade, Central Drug House, Mumbai), Dichloromethane (Ranbaxy Fine Chemicals, New Delhi), *n*-hexane (BDH, Mumbai), light liquid paraffin (Rankem, New Delhi). All chemicals and reagents used in the study were of analytical grade.

**Methods**

*Preparation of microcapsules*

All microspheres were prepared by the w/o/o double emulsion solvent diffusion technique. Weighed amounts of ethyl cellulose and salbutamol sulphate were dissolved in 5 mL of a mixture of acetonitrile and dichloromethane (1:1). The initial w/o emulsion was stirred at 500 rpm for 3-5 min. The w/o primary emulsion was then slowly added to light liquid paraffin containing 0.5% span 80 as a oil soluble surfactant with constant stirring for 2.5 h. Measured volume of *n*-hexane was added to harden the formed microspheres and the stirring was further continued for 30 to 60 min. The prepared microspheres were collected and washed several times with *n*-hexane and finally dried at room temperature. Different ethyl cellulose: salbutamol sulphate ratios (1:1, 1:2, 1:3 and 1:4) were used in order to investigate the effect of polymer/drug ratio on release and physical characterization of microspheres. The effect of stirring speed (600, 800 and 1000 rpm) and the volume of processing medium, i.e. light liquid paraffin (50, 100 and 200 mL) on microspheres characteristics were investigated.

*Physical haracterization of microspheres*

Size distribution was determined by sieving the microparticles using a nest of standard BSS sieves (36, 44, 25) as well as by optical microscopy and SEM study.

*Drug entrapment efficiency*

This test was done according to the method described elsewhere (6). A weighed quantity of microspheres equivalent to 100 mg of the pure drug were crushed into powder and added to 100 mL phosphate buffer (pH 7.4). The resulting mixture was kept stirring under magnetic stirrer for 2 h. The solution was then filtered through Whatmann filter paper. One milliliter of this stock solution was diluted using phosphate buffer (pH 7.4) and analyzed spectrophotometrically for salbutamol sulphate content at 276 nm. The drug entrapment efficiency was determined using the 

$\mathrm{Encapsulation}=\frac{\mathrm{experimental drug content}}{\mathrm{Theoretical drug content}}\times 100$

*Scanning electron microscopy (SEM)*

For morphology and surface characteristics, prepared microspheres were coated with gold in an argon atmosphere. The surface morphology of the microspheres was then studied by scanning electron microscope (Hitachi S-3600N Scanning Electron Microscope, Japan). 

*Fourier transform infrared spectroscopy (FT-IR)*

Drug-polymer interactions were studied by FT/IR spectroscopy (10). The spectra were recorded for pure drug and drug-loaded microspheres using FT-IR (Perkin Elmer, Model No. 883). Samples were prepared in KBr disks (2 mg sample in 200 mg KBr). The scanning range was 400-4000 cm^-1^ and the resolution was 2 cm^-1^. 

*Differential scanning calorimetry (DSC)*

The DSC analysis of pure drug and drug-loaded microspheres were carried out using a Diamond DSC (Perkin Elmer, USA) to evaluate any possible drug-polymer interaction. The analysis was performed at a rate 5.00ºC/min from 50°C to 200ºC temperature range under nitrogen flow of 25 mL/min (10, 11). 

*X-ray powder difftactometry (X-RD)*

X-ray powder diffractometry was carried out to investigate the effect of microencapsulation process on crystallinity of drug, as described previously (10). Powder X-RD patterns were recorded on Rigaku (Model-MenifleX, Japan) using Ni-filtered, Cuk α radiation, a voltage of 30 kV and a current of 25 mA. The scanning rate employed was 2 degrees/min, over 4° to 40° diffraction angle (2θ) range. The X-RD patterns of drug powder and drug-loaded microspheres were recorded. 

*In-vitro drug release study*

The *in-vitro *release study (5, 11-13) of the microsphere was carried out using USP basket-type dissolution test apparatus. A weighed quantity of the microspheres was introduced into the basket, the dissolution chamber was filled with 900 mL of phosphate buffer of pH 7.4 and the whole system was stirred at 100 rpm and maintained at constant temperature (37 ± 1°C). At specific time intervals, 2 mL of the sample were withdrawn and replaced by an equal volume of fresh pre-warmed dissolution medium. After suitable dilution, the samples were analyzed at 276 nm using Hitachi U-2001 UV-Visible spectrophotometer. The concentrations of salbutamol sulphate in samples were corrected to compensate the drug loss during sample withdrawal, using the equation proposed by Hayton and Chen. 

*Release kinetics*

Data obtained from *in-vitro *release studies were fitted to various kinetic equations to find out the mechanism of drug release from the ethyl cellulose microsphere. The kinetic models used were: 

Q_t_ = K_0_ . t (zero-order equation) (14) 

ln Q_t _= ln Q_0_ - k_1 _. t (first-order equation) (15) 

Q_t_ = K . S. t^1/2^ = k_H_ . t^1/2 ^(Higuchi equation based on Fickian diffusion) (16) 

where *Q*_t _is the amount of drug release in time t, Q_0_ is the initial amount of drug in the microsphere, S is the surface area of the microcapsule and k_0_ , k_1_ , and k_H_ are rate constants of zero order, first order and Higuchi equations, respectively. In addition to these basic release models, there are several other models as well. One of them is Korsenmeyer-Peppas equation (power law) (17).

M_t _/ M_∞_=k . t^n^

where M_t_ is the amount of drug release at time t and M_∞_ is the amount release at time t = ∞, thus M_t_ / M_∞_ is the fraction of drug released at time t, k is the kinetic constant, and n is the diffusion exponent which can be used to characterize both mechanism for both solvent penetration and drug release. Determining the correlation coefficient assessed fitness of the data into various kinetic models. The rate constants for respective models were also calculated from slope.

*Statistical analysis*

The data obtained from the particle size, encapsulation efficiency and release rate determination studies of salbutamol sulphate microspheres were analyzed statistically with ANOVA and t-test to evaluate its significance.

## Results and Discussion

The primary requirement of this method to obtain microspheres is that the selected solvent system for polymer be immiscible with non-aqueous processing medium (18). Acetonitrile is a unique organic solvent which is polar, water miscible and oil immiscible. When acetonitrile alone is used as a solvent along with oil as the processing medium, it does not ensure the formation of primary emulsion of the aqueous phase in the polymer solution. Immediately on mixing, the water miscibility of acetonitrile brought about the precipitation of the polymer (ethyl cellulose). Hence, a non-polar solvent, namely dichloromethane was included with acetonitrile to decrease the polarity of the polymer solution. The optimal proportion of dichloromethane and acetonitrile was found to be 1:1, which enabled emulsion formation and yielded good free flowing microspheres. No surfactant was used to stabilize the primary emulsion, since ethyl cellulose has the additional property of stabilizing w/o emulsion. Span 80, an oil miscible nonionic surfactant was used to stabilize secondary emulsification process.

*Mean particle size*

The particle size of the microspheres was in the range of 271 μm to 416 μm (Table 1). It was observed that when polymer amount increased, particle size of the microspheres increased (P < 0.05). When the stirring speed was increased from 600 to 800 rpm, particle size increased (P < 0.05) as shown in Table 1; it may be due to an increase in viscosity of the polymer solution. Also, when the volume of processing medium was decreased, polymer and drug concentration increased. As a result of the increase in the polymer concentration, microspheres particle size increased (P < 0.05) as shown in Table 1.

**Table 1:** Physical characterization of microspheres

**Batch Code**	**Drug/Polymer** **ratio**	**Volume of processing medium (mL)**	**Stirring speed** **(rpm)**	**Particle size** **(μm)**	**Entrapment efficiency (%)**
F1	1:1	50	800	416 ± 3.7	67.3 ± 1.17
F2	1:2	50	800	371 ± 2.1	76.6 ± 0.45
F3	1:3	50	800	297 ± 1.4	62.5 ± 1.25
F4	1:4	50	800	271 ± 1.3	64.9 ± 1.37
F2a	1:2	50	600	397 ± 2.4	61.7 ± 1.33
F2b	1:2	50	1000	271 ± 1.8	63.1 ± 0.98
F2c	1:2	100	800	297 ± 1.8	67.4 ± 0.93
F2d	1:2	200	800	281 ± 0.9	55.7 ± 0.78

* All values are expressed as Mean ± SD, n=3

*Scanning electron microscopy*

**Figure 1:**
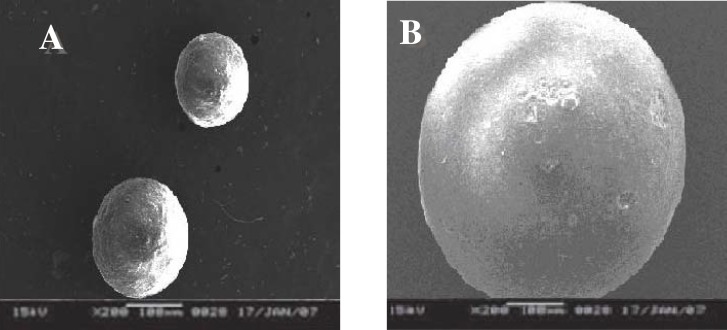
Surface topography of salbutamol sulphate-loaded ethyl cellulose microspheres. A: blank microspheres; B: drug-loaded microspheres

**Figure 2:**
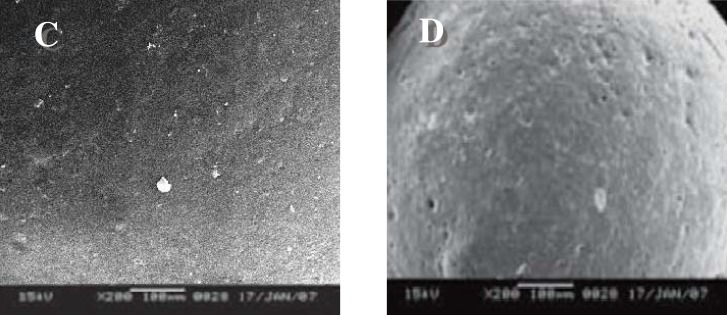
Surface topography of salbutamol sulphate-loaded ethyl cellulose microspheres. C: drug-loaded microspheres before dissolution; D: drug-loaded microspheres after dissolution

SEM study shows (Figure 1) that particles were spherical in shape and exhibited porous surfaces. The surface of the drug loaded microspheres manifested the presence of drug particles as compared to blank microspheres (Figure 1). Surface study of the microspheres after release study showed bigger pores suggesting that the drug is released though pores and the mechanism of drug release was diffusion controlled (Figure 2).

*FT-IR analysis*

**Figure 3:**
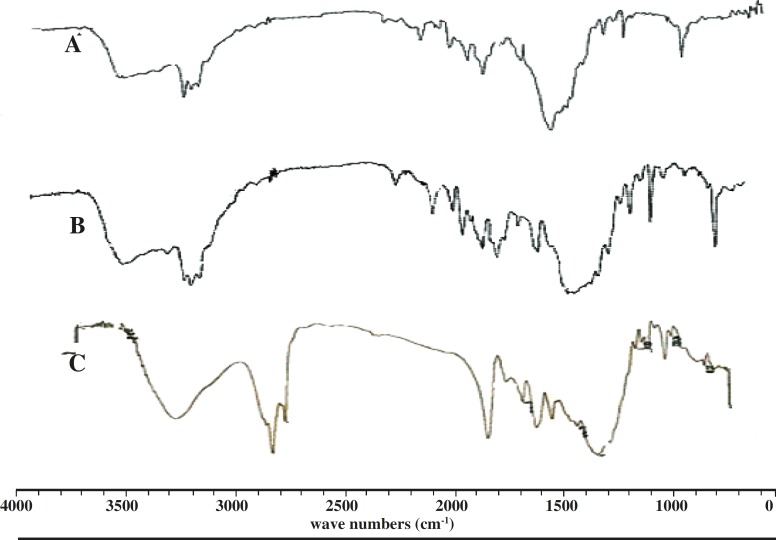
FT-IR spectra of pure drug salbutamol sulphate (A), drug-loaded microspheres (B), blank ethyl cellulose microspheres (C).

The IR-spectrum of salbutamol sulphate showed sharp peaks at 1100 cm^-1^ (C-O stretching) and at 1500 cm^-1^ for O-H bending. The identical peaks were also present in salbutamol sulphate-loaded ethyl cellulose microspheres. All the identical peaks were also obtained in the microspheres, confirming their compatibility (Figure 3).

*Differential scanning calorimetric studies*

**Figure 4:**
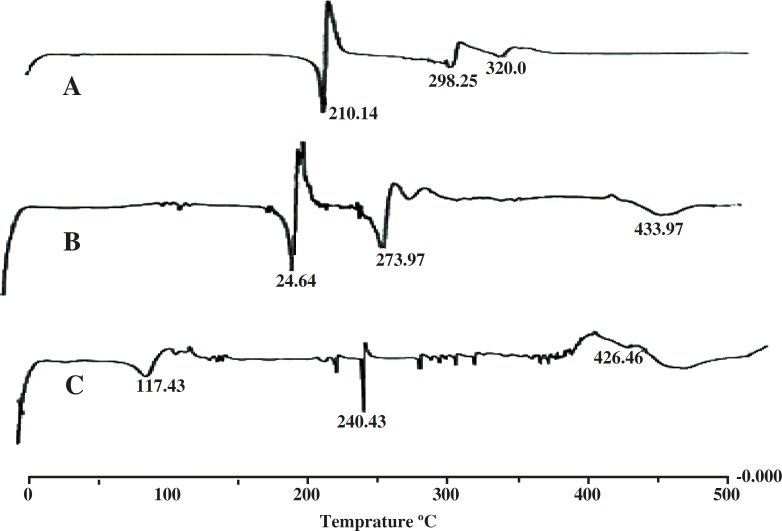
DSC thermogram of pure drug salbutamol sulphate (A), drug-loaded microspheres (B), blank ethyl cellulose microspheres (C).

The DSC graphs of salbutamol sulfate and salbutamol sulfate-loaded microspheres are presented in Figure 4. The DSC curve of pure drug shows a sharp endothermic melting peak with the onset of about 200ºC reaching maximum at 216ºC and the same was also observed in the drug-loaded microspheres. Other endothermic peaks are found at 298.25ºC and 328.99ºC. The DSC curve of salbutamol sulfate-loaded microspheres shows broad peaks from 290 to 310°C which is due to the physicochemical binding of the drug with the polymer structure. 

*X-RD studies*

X-RD technique has been extensively utilized along with DSC to study the physical state of the drug in the polymer matrix. The crystalline nature of the drug was clearly demonstrated by its characteristics X-RD pattern containing well define peaks between 2 theta of 22° to 40°. 

However, drug-loaded microspheres exhibited characteristic diffraction pattern, which was less intense as compared to pure drug. The presence of diffract gram which was much decreased in the drug-loaded microspheres indicated that drug present in the polymer matrix in crystalline state and the presence of polymer further decreased the crystallinity of the pure drug. 

*Effect of formulation and processing variable on the characteristics of microspheres*

Effect of various processing and formulation parameters on drug entrapment efficiency of microspheres are shown in the Table 1. The highest entrapment efficiency was achieved by increasing drug-polymer ratio from 1:2 to 1:4 (P > 0.05). With further increase in drug to polymer ratio from 1:2 to 1:1, a significant decrease in entrapment efficiency was observed. This difference was significant (P < 0.01) for both the formulations at different polymer to drug ratio. This suggests that higher concentration of drug decreases encapsulation efficiency of salbutamol sulphate due to higher concentration gradient, resulting in the drug diffusion out of the polymer/solvent droplets to the external processing medium. 

The volume of processing medium significantly influenced the entrapment efficiency of the drug-loaded microspheres (Table 1). As the volume of processing medium was increased from 50 mL to 100 mL and to 200 mL, the entrapment efficiency was further decreased from 76.6% to 55.7%, respectively. The reason may be the higher amount of drug extraction into the processing medium, resulting in lower entrapment efficiency. However, the difference is statistically significant (P < 0.05) when the volume was increased from 50 to 100 mL, and (P < 0.01) as the volume increased from 100 to 200 mL. 

The entrapment efficiency was also influenced with changing the stirring speed of the secondary emulsification process. The highest entrapment efficiency was observed with the stirring speed of 800 rpm. The change of stirring speed from 800 rpm to 600 and 1000 rpm significantly decrease the entrapment efficiency (P > 0.05). 

*Drug release behaviour*

**Figure 5:**
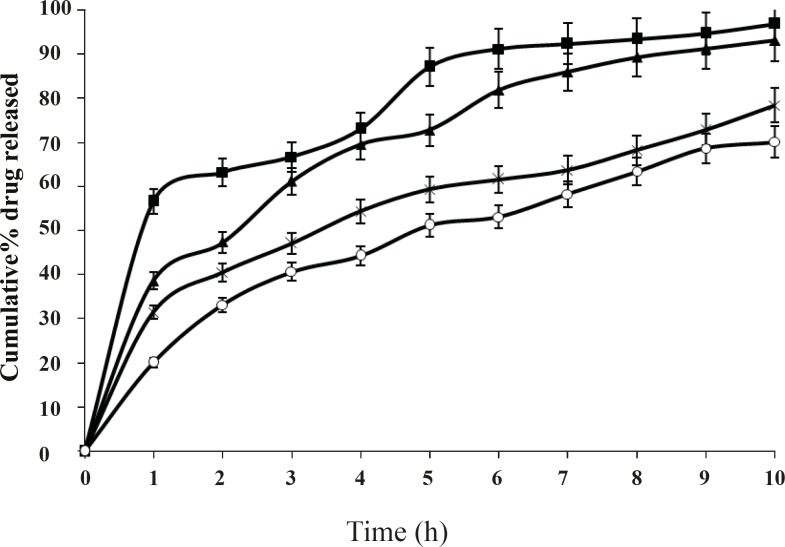
Effect of drug to polymer ratio on *in-vitro *drug release from microspheres containing different drug to polymer ratios 1:1, (-■-), 1:2,(-×-), 1:3,(-▲-), 1:4,(-o-); Mean ± SD; *n=*3

When the concentration of the polymer in the system increased the release rate of salbutamol sulphate decreased. The difference was also significant (P < 0.01) for 8 h. It is also observed that *in-vitro *release of salbutamol sulphate from ethyl cellulose microspheres exhibited initial burst release. This may be due to the presence of drug particles adhered on the surface of the microspheres. The burst effect of salbutamol sulphate released from the microspheres decreased significantly when the drug to polymer ratio was increased from 1:1 to 1:2, 1:3, 1:4, etc (P < 0.01). The best *in-vitro *sustained drug release was observed in the formulation F2, when drug to polymer ratio was 1:2. The release profiles of different batches were illustrated in Figure 5. 

These observations could be attributed to the fact that an increase in the polymer solution viscosity has produced microspheres with reduced porosity due to the thickening of the polymer wall. It is understood that higher polymer concentration results in a longer diffusional path length, so drug release is extended. The thick polymeric barrier slows the entry of surrounding dissolution medium in to the microspheres and hence less quantity of drug leaches out from the polymer matrices of the microspheres exhibiting extended release.

**Figure 6:**
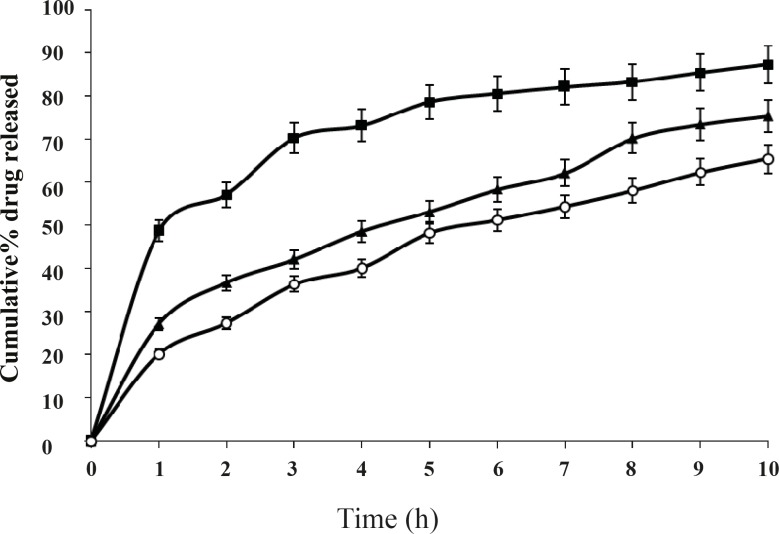
Effect of stirring speed on *in-vitro *drug release from microspheres carrying drug to polymer ratio 1:2 at stirring speed 600 rpm (-■-), 800 rpm (-▲-) and 1000 rpm (-o-); Mean ± SD; *n=*3

The change of stirring speed of secondary emulsification process also influenced the drug release from microspheres as shown in Figure 6. 

**Figure 7:**
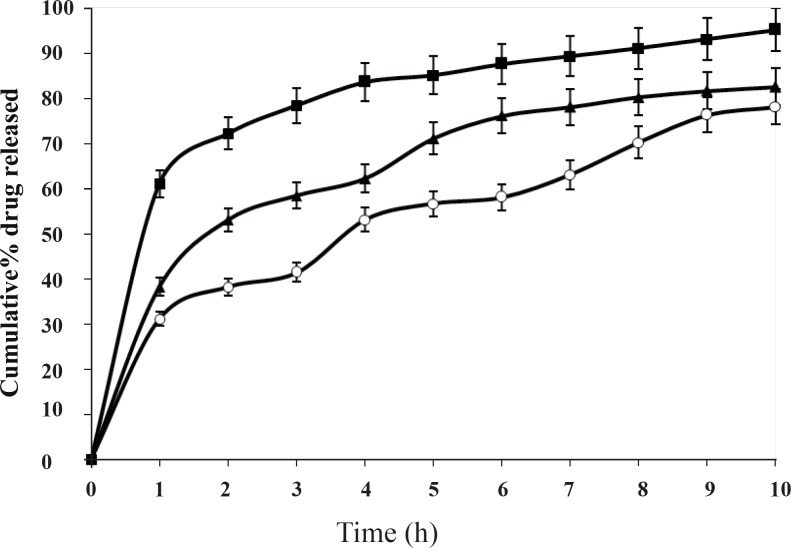
Effect of volume of processing medium on *in-vitro *drug release from microspheres carrying drug to polymer ratio 1:2, 50 mL (-o-), 100 mL (-▲-), 200 mL (-■-); Mean ± SD; *n=*3

This difference was statistically significant (P < 0.05) for the formulations prepared at different stirring speed. It is observed that amount of drug release increases at the lowest stirring speed. This may be due to the adherence of drug particles on the surface of the polymeric matrices. Whereas, at higher stirring speed drug release further decreases. However, the best release was observed with the formulation F2, at the stirring speed of 800 rpm. Volume of processing medium also influenced the drug release to a large extent as shown in the Figure 7. This difference was significant (P < 0.05) for 8 h. It is observed that larger volume of processing medium shows higher amount of drug release as compared to lower volume of processing medium. This may be due to the fact that drug particles move freely to the surface of the polymeric matrix at larger volume of processing medium during solvent evaporation process resulting faster rate of drug release. The best release was observed with the formulation F2, when the volume of processing medium was 50 mL, as shown in Figure 7. 

*Release kinetics*

The release mechanism of salbutamol sulphate from various formulations was successfully determined by comparing their respective correlation coefficient. The best fit with the highest correlation coefficient was found in Higuchi, followed by first-order and zero-order kinetics. It revealed that the drug release from ethyl cellulose microspheres was diffusion controlled. The data obtained were also fitted in Korsemeyer-Peppas model in order to find the n value. The n value of different microspheres prepared by altering drug to polymer ratio lies in between 0.261 to 0.52, indicating that the mechanism of drug release was diffusion controlled (Fickian diffusion). 

## Conclusion

Salbutamol sulphate was successfully encapsulated into ethyl cellulose microspheres using w/o/o emulsion solvent evaporation method. The encapsulation efficiency was also influenced with changing the stirring speed of the secondary emulsification process. The *in-vitro *release of salbutamol sulphate from ethyl cellulose microspheres exhibited initial burst effect which was due to the presence of drug particles on the surface of the microspheres. The initial burst effect may be attributed as a desired effect to ensure initial therapeutic plasma concentration of the drug. Factors such as drug to polymer ratio, volume of processing medium and stirring speed of secondary emulsification process govern the drug release from the microspheres. Evaluation of the release kinetic data showed the highest correlation in the Higuchi plot, indicating that the drug release from ethyl cellulose microspheres was diffusion controlled.
